# Sense of coherence over time for parents with a child diagnosed with cancer

**DOI:** 10.1186/1471-2431-12-79

**Published:** 2012-06-21

**Authors:** Ingrid Bergh, Maria Björk

**Affiliations:** 1 School of Life Sciences, University of Skövde, Box 408, 541 28, Skövde, Sweden

## Abstract

**Background:**

When a child is diagnosed with childhood cancer this creates severe stress in the parents. The aim of the study was to describe the sense of coherence and its change over time in a sample of parents of children diagnosed with cancer.

**Methods:**

The Swedish version of SOC (29 items) was used to measure the parents’ (n = 29) sense of coherence. Data were collected at four time-points: Time-point 1 at the time of diagnosis; time-point 2 during the treatment; time-point 3 after the child had completed their treatment and time-point 4 when the child had been off treatment for some years or had died.

**Results:**

The results showed that SOC in the investigated population is not stable over time. The parents decreased in total SOC between time-points 1, 2 and 3. Mothers had significantly weaker total SOC score including the components Manageability and Meaningfulness at time-points 1 as well time-point 2 compared to the fathers. However, for the component Comprehensibility no significant differences were shown between mothers and fathers. This study indicates that mothers’ and fathers’ SOC scores change over time during the child’s cancer trajectory. However, the pattern in these changes varies between mothers and fathers.

**Conclusions:**

This study indicates that mothers and fathers may have different support needs during their child’s cancer trajectory.

## Background

In the developed world cancer affects 1 in 600 children before the age of 15 [1]. In Sweden every year 300 children aged 14 and under are diagnosed with cancer [2]. Even though the survival rate in Sweden exceeds 75% [3] childhood cancer is a life-threatening condition for the child and a severe trauma for the entire family [4-6]. The diagnosis dramatically changes life for the entire family [7] and brings severe distress to most parents [8]. They can suffer for an extended time from strain arising from their child’s illness. Severe stress reactions could be expected in a significant proportion of parents during the years following diagnosis [9].

A person’s sense of coherence (SOC) reflects their orientation to life and the extent to which they experience life as comprehensible, manageable and meaningful [10]. For individuals with a strong SOC life is perceived as comprehensible, manageable and meaningful and they probably cope successfully with stressful life events. On the other hand, those with a weak SOC experience life as more chaotic, unmanageable, and meaningless and stressful life situations can be experienced as overwhelming. Antonovsky [10] states that adults’ SOC is a deep-rooted attitude to life. SOC develops during childhood and early adulthood and stabilizes at around 25-30 years of age. There can however be temporary changes around the individual’s mean score but this is a transient condition [10]. In contrast, several studies have shown SOC to be a flexible construct responding to changes in life situation [11,12] and may also reflect symptoms of depression and anxiety [13]. On the other hand, people with high SOC scores seem to maintain their stability independent of events [14]. Parents of children diagnosed with diabetes, epilepsy or psychiatric/nervous problems were about 2-5 times more likely to have a lower SOC than those of children without such diagnoses [15]. It has also been shown that high SOC scores in parents of children with Down’s syndrome reduce self-perceived stress [16]. It is important to pay attention to the parents’ wellbeing as they play an important role in their children’s lives. The child greatly needs their parents as a secure base, especially when they are not feeling well and during hospital visits [17-19]. Therefore, the aim of this study was to describe sense of coherence over time in a sample of parents with a child newly diagnosed with cancer.

## Methods

### Context

The study was performed at a University Hospital in the south of Sweden with a catchment area of approximately 1.8 million people. The paediatric oncology unit comprises a 16-bed ward, a day care ward and a consultant. Approximately 60 children newly diagnosed with cancer are admitted each year.

### Sample

During a ten month period parents with a child newly diagnosed with cancer were consecutively asked to participate in the study. Inclusion criteria were 1) that the child was under the age of 13 when diagnosed; 2) that the diagnosis was a first time one; 3) that the parent could speak and understand Swedish and finally 4) that the child’s treatment (surgery in combination with chemotherapy/radiation, or chemotherapy or radiation alone) was initiated within one month of diagnosis. During the inclusion period 44 children were diagnosed with cancer. Twenty seven of these fulfilled the inclusion criteria and were asked to participate. After refusals to participate, 29 parents (mothers, n = 17; fathers, n = 12) agreed to take part in the study. The diagnoses for the children were: leukaemia (n = 9), brain tumour (n = 4), and solid tumour (n = 4). A description of the participants is shown in Table 1.

**Table 1:** Description of the participants

**Number of participants (n)**	**29**
Mothers	17
Fathers	12
Mother’s age range (median)	30-42 (35)
Father’s age range (median)	31-45 (35)
Single-parent family	2
Parents born outside Sweden	4
Parental education (n)	
Nine-year compulsory	2
Upper secondary school	14
College	6
University studies	7
Parent working	2
On sick leave or unemployed	27
Number of children within the family	
Range	1-5
Median	2

### Instrument

The Swedish version of SOC, which appears in the Swedish edition of “Unravelling the mystery of health” [20], was used to measure the parents’ sense of coherence. The questionnaire used comprises 29 items, rated on a seven point Likert scale (ranging between two extremes i.e. “never” to “very often”), and reflects the components of comprehensibility, manageability and meaningfulness. Thirteen items are negatively stated and must be reversed before they are analysed. The possible scores range from 29 to 203.

### Data collection

Two designated nurses gave written information to parents eligible for the study at a suitable time after the diagnosis. After the parent had given written consent to participate, they were contacted by the investigator. The dates and places for data collection were decided in agreement with the parents. The time-points for data collection were: Time-point 1: at the time of diagnosis; time-point 2, during the treatment; time-point 3, after completion of treatment; time-point 4, when the child had been off treatment for some years, or had died. The numbers of parents participating in the data collection varied at the different time-points due to adverse events (Table 2).

**Table 2:** Numbers of respondants at the time-points

	***Time-point***			
	***1***	***2***	***3***	***4***
Mothers	17	11	11	7
Fathers	12	8	9	6
Total	29	19	20	13

Time-point 1 = Months after diagnosis (Median = 2; range = 1-5).

Time-point 2 = Months after diagnosis (Median = 9; range = 7-15).

Time-point 3 = Months after diagnosis (Median = 22; range = 13-36).

Time-point 4 = Months after diagnosis (Median = 87; range = 24-105).

### Statistical analysis

In no cases were more than 10% of the responses missing [21] but when that occurred, median substitution was performed by replacing missing data with the median value within the components of SOC (i.e. Comprehensibility, Manageability and Meaningfulness). Since data characteristics did not meet the criteria for parametric analysis (i.e. normally distributed, interval or ratio data), non-parametric tests were used. Mann-Whitney's *U*-test was used when comparing independent groups (mothers and fathers) and Wilcoxon signed-rank test for pair-wise comparison of dependent groups (SOC and its components at different time-points) [21]. Values of *p*-value < 0.05 two-tailed were considered statistically significant for all tests. The results were analysed in PASW SPSS 18.0.

### Ethical considerations

At inclusion, written informed consent was obtained from each parent. This was repeated orally before the two following occasions and finally a new written informed consent was obtained before the last occasion. This research was formally approved by the Research Ethics Committee of the Medical Faculty, Lund University, Sweden, (LU 476-01; 2009/127), and followed the principles of research ethics approved by the Swedish Medical Research Council [22]. This means that the four ethical principles: respect for autonomy, beneficence, nonmaleficence and justice were considered [22,23].

## Results

**Figure 1:**
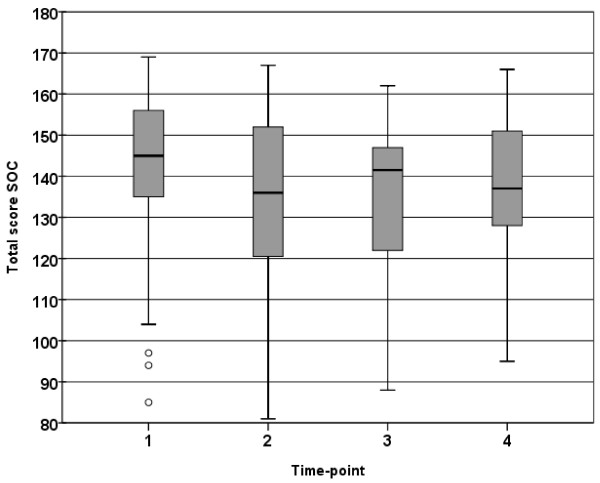
**Box plot showing the distribution (25**^**th**^**, 50**^**th**^**, 75**^**th**^**quartile) of total SOC score (total sample) at the 4 time-points**.

The parents decreased in total SOC between time-points 1 and 2 (*p* = 0.002), this level remained constant until time-point 3 (*p* = 0.005). There were no significant difference between time-point 1 and 4 (Figure 1).

**Figure 2:**
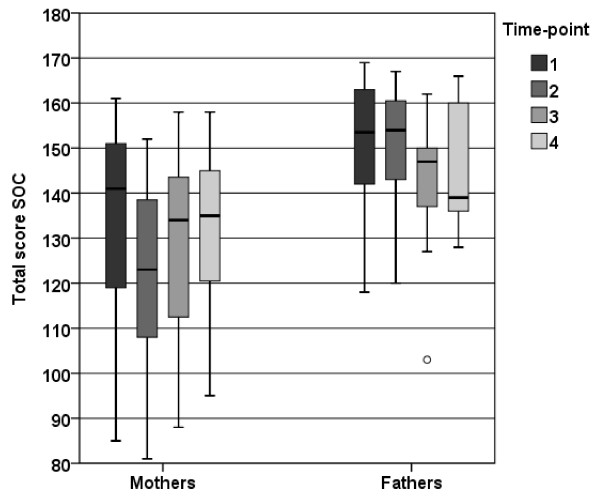
**Box plot showing the distribution (25th, 50th, 75th quartile) of total SOC score for mothers and fathers respectively at the 4 time-points**.

There were significant differences between total SOC of fathers and mothers at time-points 1 (p = 0.034) and 2 (*p* = 0.009). However, no significant difference was observed at time-points 3 and 4 (Figure 2).

Mothers’, but not fathers’, total SOC decreased (*p* = 0.016) between time-points 1 and 2. However, fathers’ SOC showed a significant decrease between time-points 1 and 3 (*p* = 0.012), but this decrease had gone by time-point 4 (*p* = 0.058) (Figure 2).

**Figure 3:**
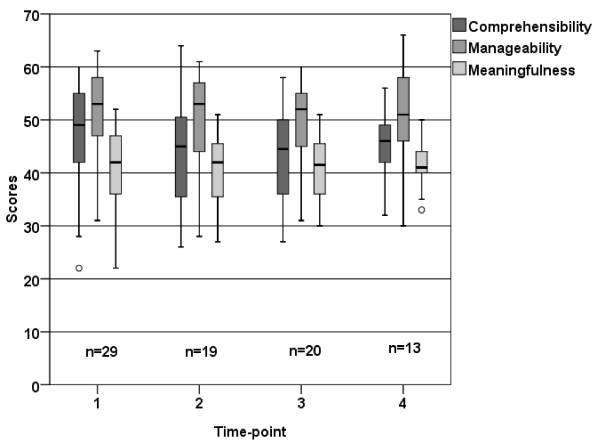
**Box plot showing the distribution (25th, 50th, 75th quartile) of scores of the three components of SOC (Comprehensibility, Manageability and Meaningfulness) at the 4 time-points (total sample)**.

Overall there were a decrease in Comprehensibility (*p* = 0.017) and Meaningfulness (*p* = 0.004) between time-point 1, and 2, however, Manageability showed no differences between any of the time-points (Figure 3).

**Figure 4:**
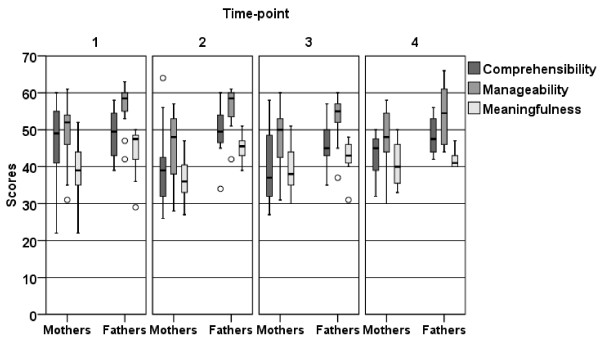
**Box plot showing the distribution (25th, 50th, 75th quartile) of the three components of SOC (Comprehensibility, Manageability and Meaningfulness) for mothers and fathers at the 4 time-points**.

There was no significant differences between mothers and fathers in Comprehensibility at all four time-points. Mothers had significantly lower Manageability than did fathers at time-point 1 (p = 0.005) and 2 (*p* = 0.009) but no difference were obtained at time-points 3 and 4. The mothers also showed a significantly lower Meaningfulness at time-point 1 (*p* = 0.018) and 2 (*p* = 0.004) compared to the fathers, no significant differences in Meaningfulness were seen at time-points 3 and 4 (Figure 4).

Mothers scored lower Comprehensibility at time points 2 (*p* = 0.040) and 3 (*p* = 0.023) compared to time point 1. They also scored Meaningfulness lower at time point 2 (*p* = 0.016) compared to time point 1, this decrease was not present at time-points 3 and 4. For fathers, no differences were obtained between the various time points in Manageability except that they scored higher at time point 3 than time point 1 (*p* = 0.017). For fathers Meaningfulness decreased between time-points 1 and 3 (*p* = 0.012), this decrease was still evident at time-point 4 (*p* = 0.027) (Figure 4).

## Discussion

This study involves a small sample of parents, too small to draw definite conclusions though some interesting results need to be discussed. The main findings were that SOC is not stable over time. Moreover, mothers had significantly weaker total SOC scores than did fathers for Manageability and Meaningfulness at time-points 1 and 2. However, for Comprehensibility no significant differences between mothers and fathers were obtained at any of the four time-points.

Results indicated a gender difference in SOC score, with fathers reporting it higher (although not statistically significantly at every time point) than mothers. This is corroborated in other studies. Eriksson and Lindström [24] found in their review of the SOC scale that men usually report a slightly higher SOC score than women. However, studies in “normal” Swedish populations have not indicated any gender differences in SOC [25,26]. This kind of inconsistency has also been reported when comparing mothers and fathers of children with intellectual disabilities [16,27].

The present work showed that SOC in the sample studied was not stable over time. Whether SOC is stable over time (in life) has been questioned in several studies e.g. [11,12,24,28]. It seems to be a flexible construct responding to changes in life situations [11,12]. In our study it also seemed that fathers’ SOC score decreased during the latter part of the child’s cancer trajectory while the opposite is true for mothers.

The results also highlight that mothers had significant weaker score than did the fathers on the components Manageability and Meaningfulness at time-points 1 and 2. Reay and co-workers [29] described mothers are often the one at home taking care of the child and in contrast fathers taking care of his job, earning money. The parent responsible for caring for the child at home often feels exhausted as they have to deal with hospital visits, siblings, child raising and the household generally. They can also feel locked in to the sick child and the home and they look forward to moments where they could think of something else and socialize with adults [30]. This may be the cause of mothers having a lower score on Manageability as well as Meaningfulness.

The total sample, especially the mothers, decreased in the component Comprehensibility between time-points 1 and 2. Diagnosis of cancer in a child undermines the vision of a long and happy life [2]. The family has to deal with the loss of a healthy child as well as to live with the uncertainty which the disease brings [31]. Björk et al. [5] reported that parents of children newly diagnosed with cancer found that the situation was unreal and they wanted to escape from it. They lost foothold in life as well as their sense of security and they became vulnerable. It seems reasonable to believe that those findings are reflected in the results from the present study.

It is therefore important for health care professionals associated with these families to pay attention to each individual mother’s and father’s experiences and needs. One useful approach is family centred care (FCC) defined as “a way of caring for children and their families within health services which ensures that care is planned around the whole family, not just the individual child/person, and in which all the family members are recognized as care recipients” [32], p. 1318. To communicate with the family as a whole can be beneficial for both the family and the staff [33].

## Conclusions

This study indicates that when a child is diagnosed with cancer parents’ SOC scores change over time. However, the pattern in these changes varies between mothers and fathers during the cancer trajectory. This also suggests that they may have different support needs during the trajectory. Therefore, future research is needed in larger samples using a longitudinal approach to explore further these parents’ SOC as well as symptoms of depression and anxiety.

## Competing interests

The authors declare that they have no competing interests.

## Authors’ contributions

MB was responsible for the study design and data collection. MB and IB conducted the data analysis and drafting of the manuscript. Both authors read and approved the final manuscript.

## Pre-publication history

The pre-publication history for this paper can be accessed here:

http://www.biomedcentral.com/1471-2431/12/79/prepub
